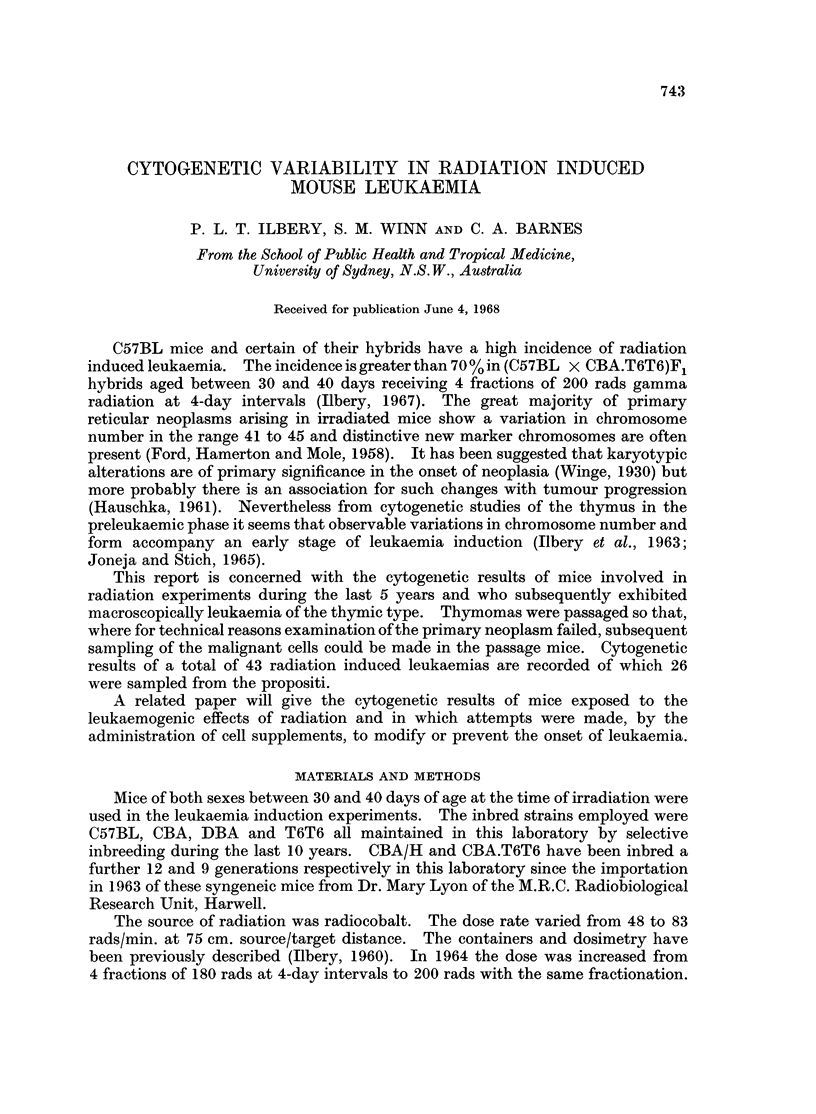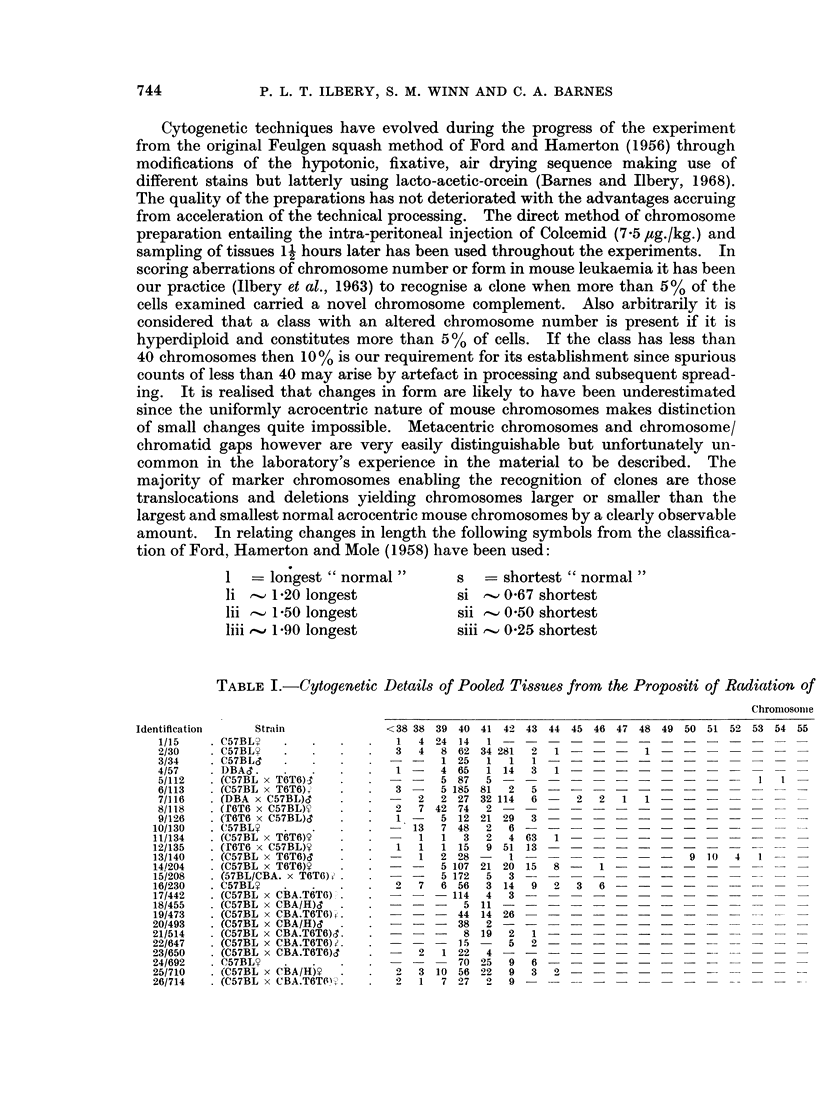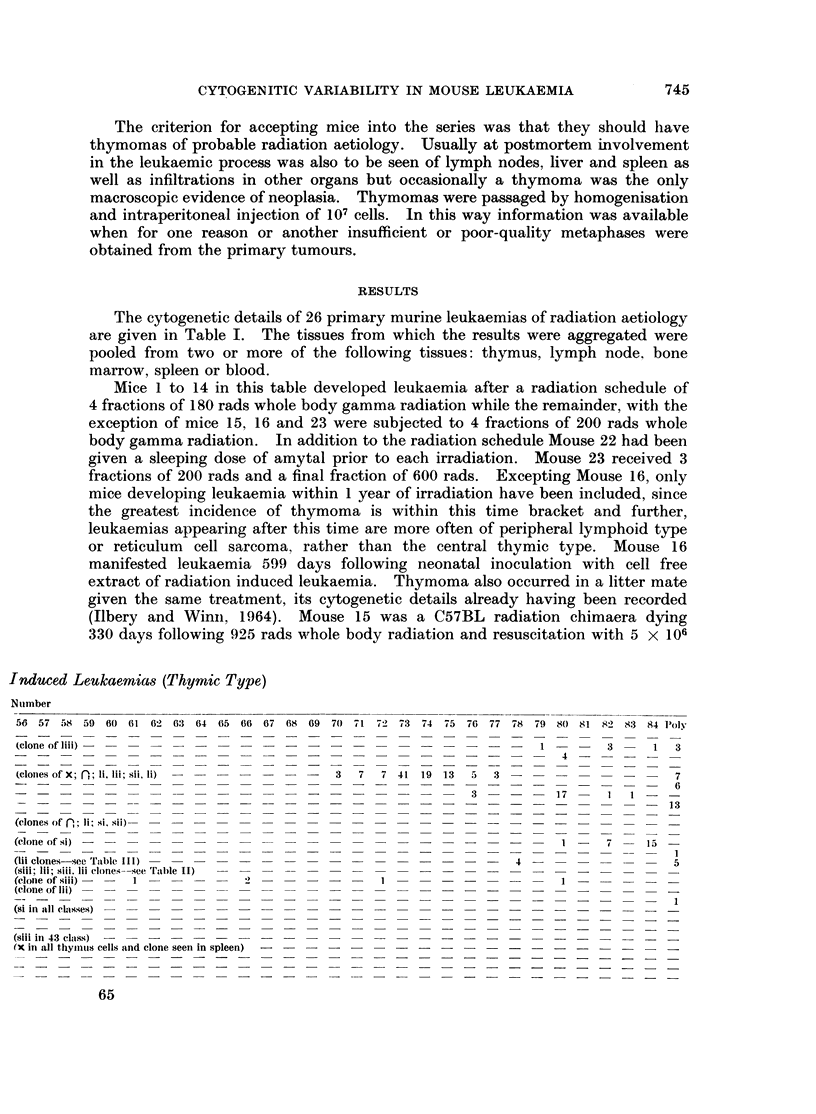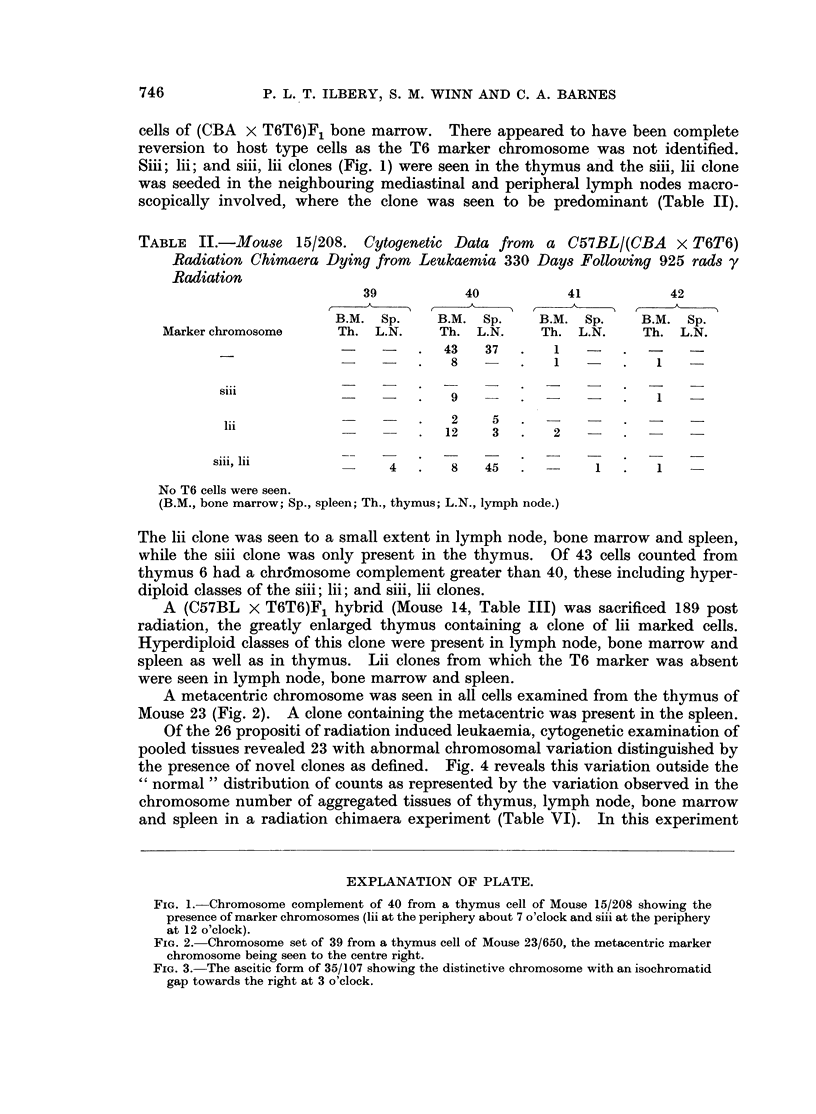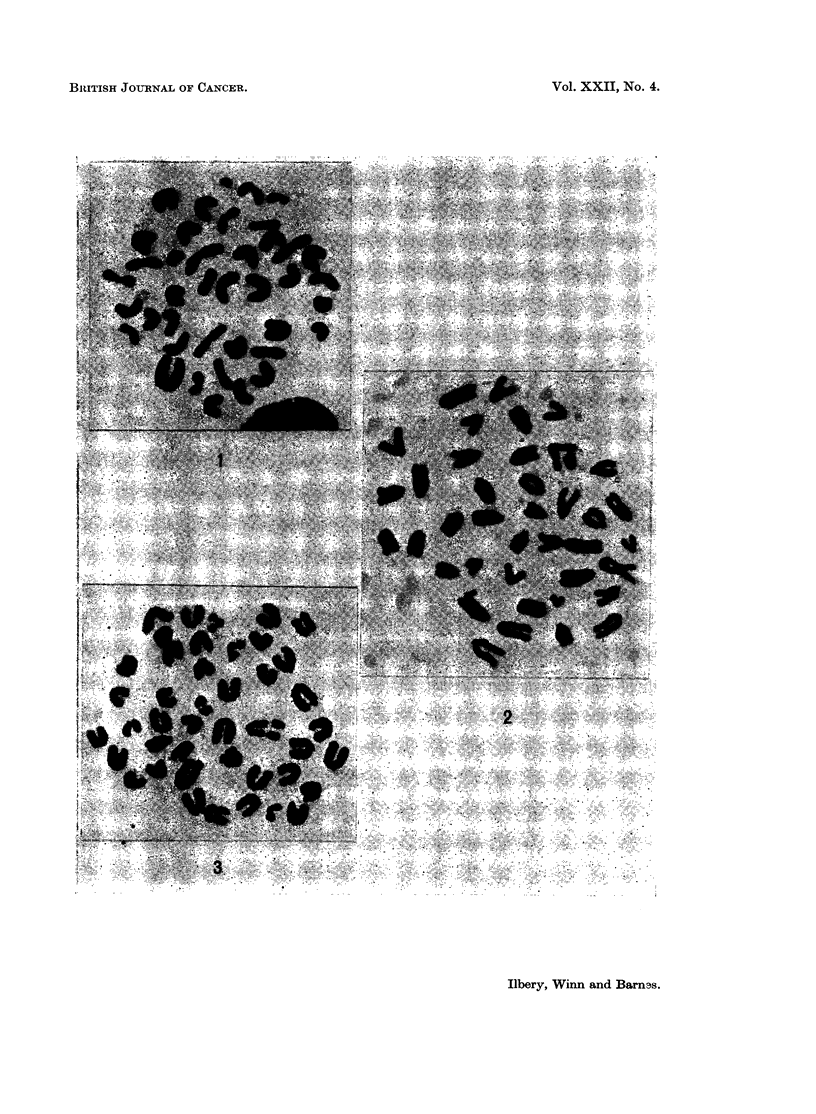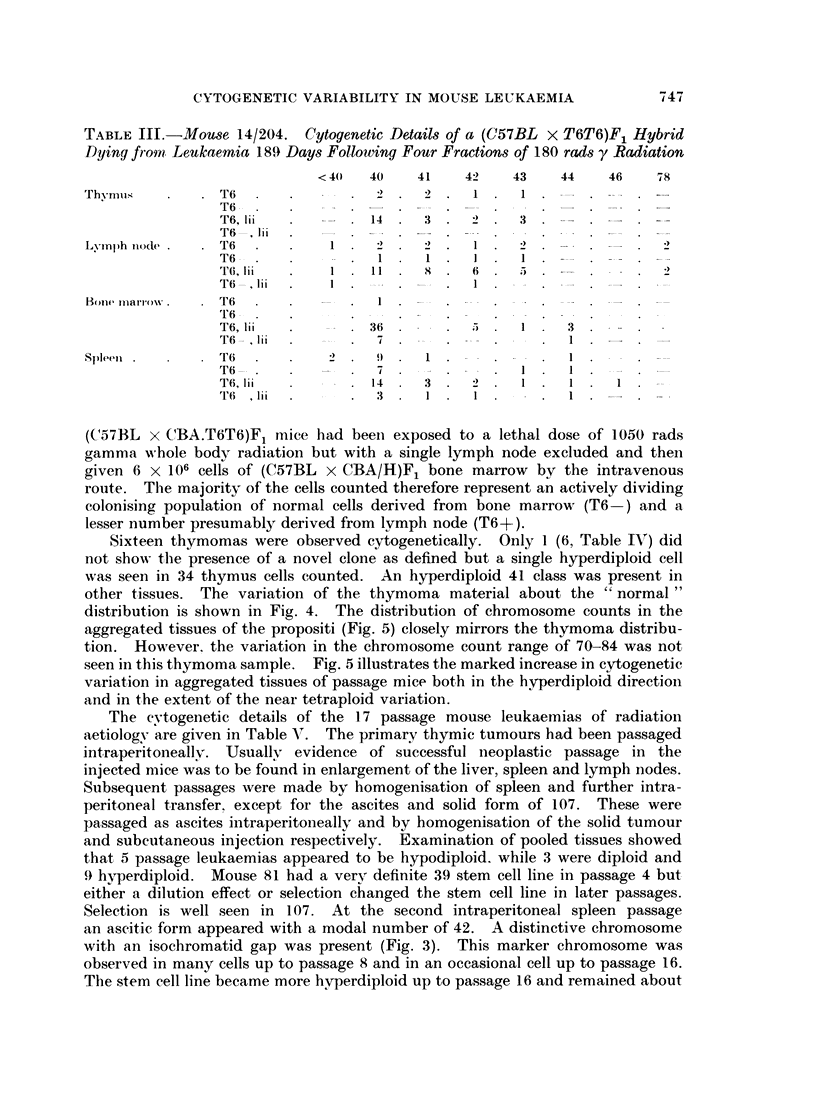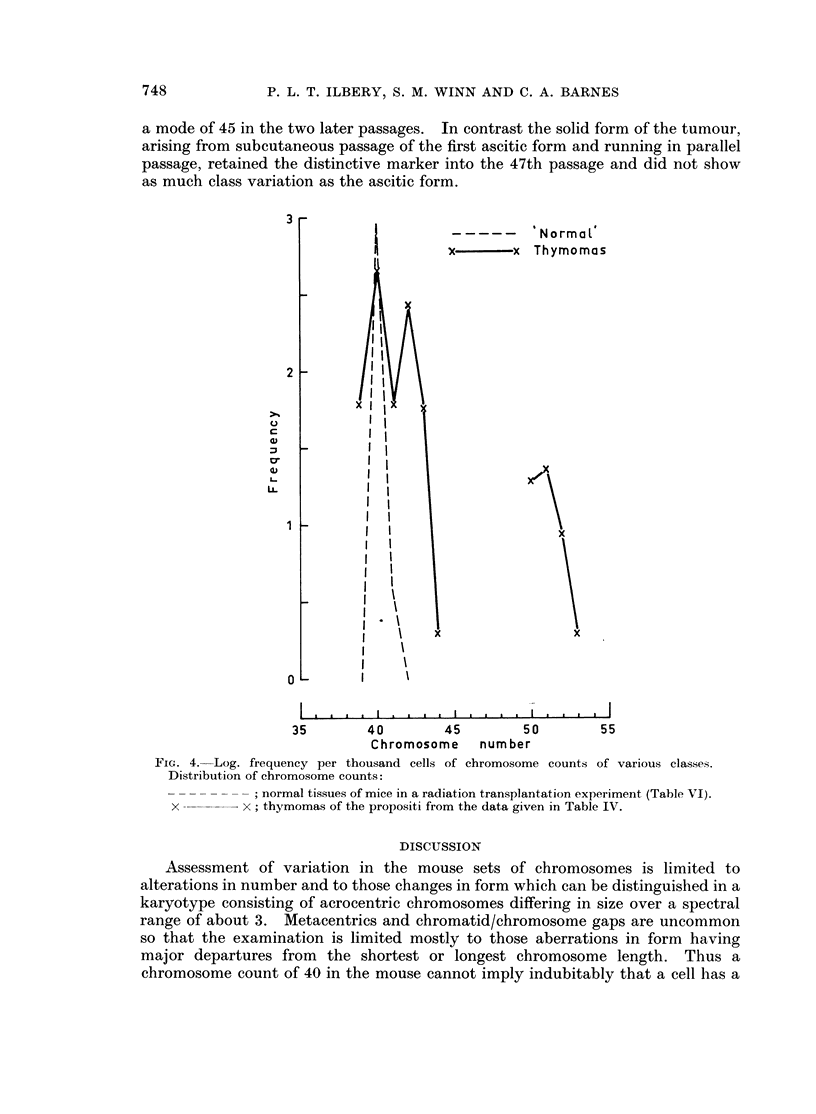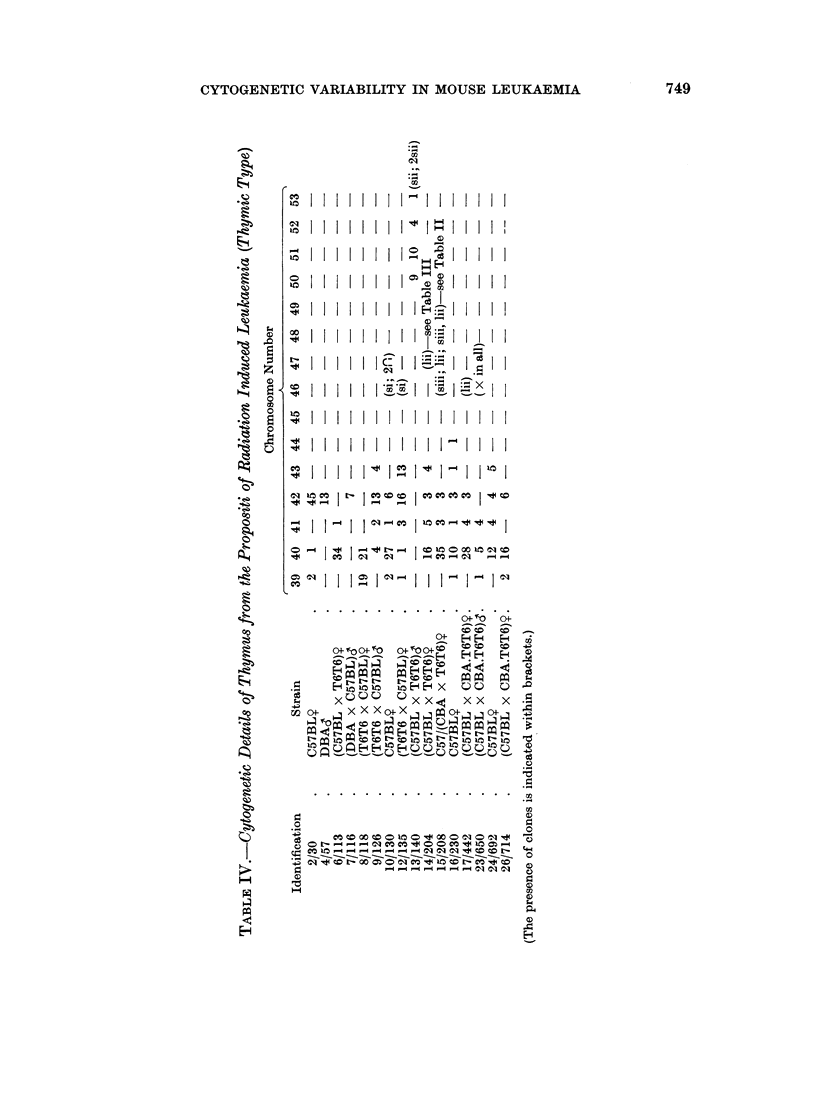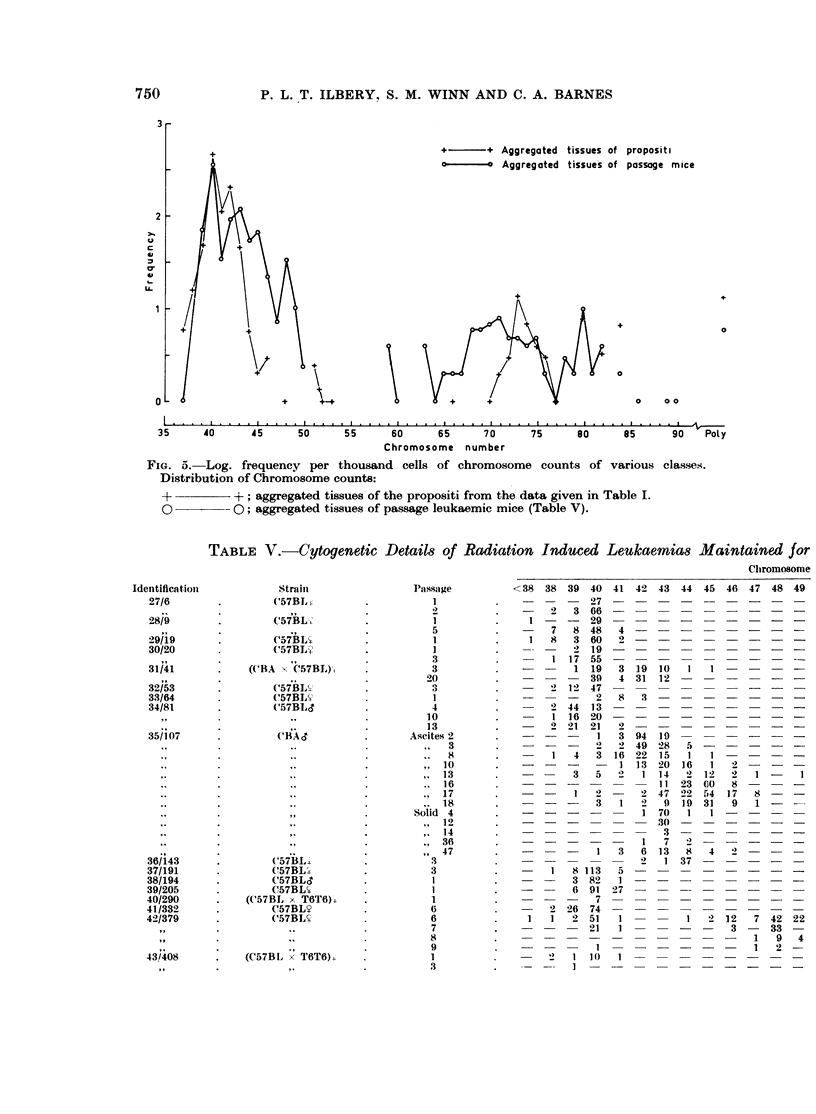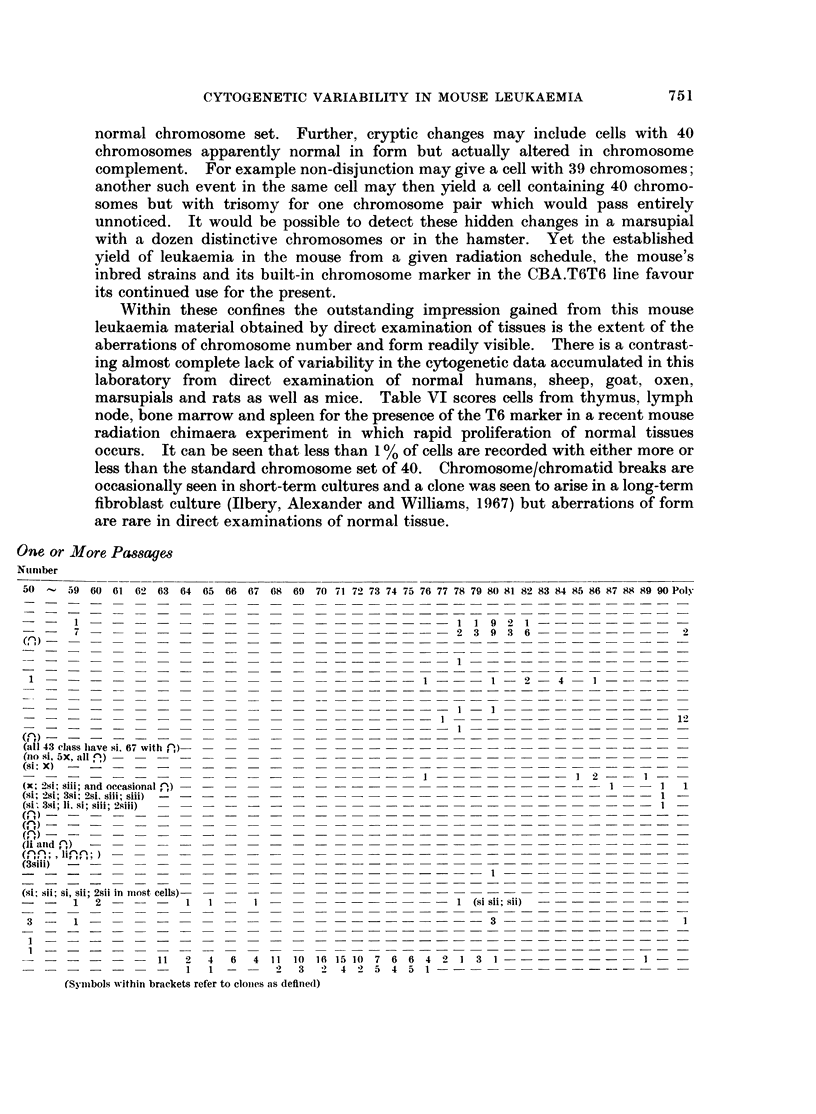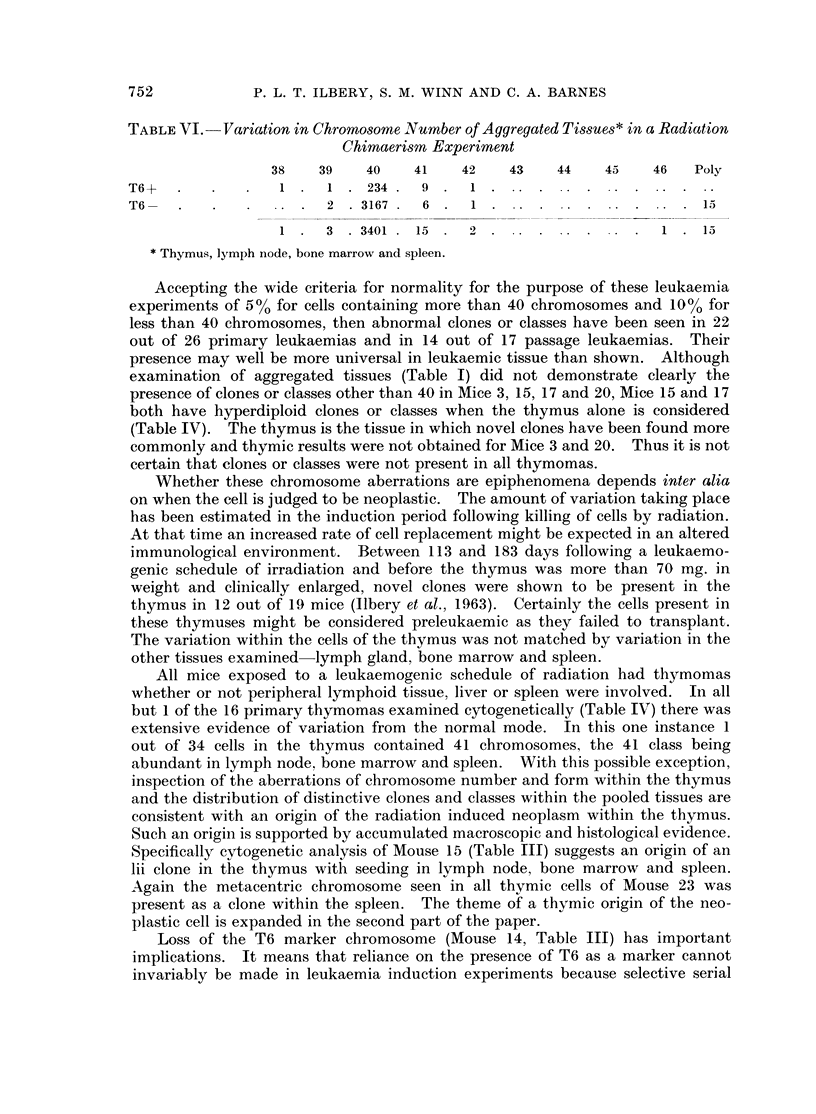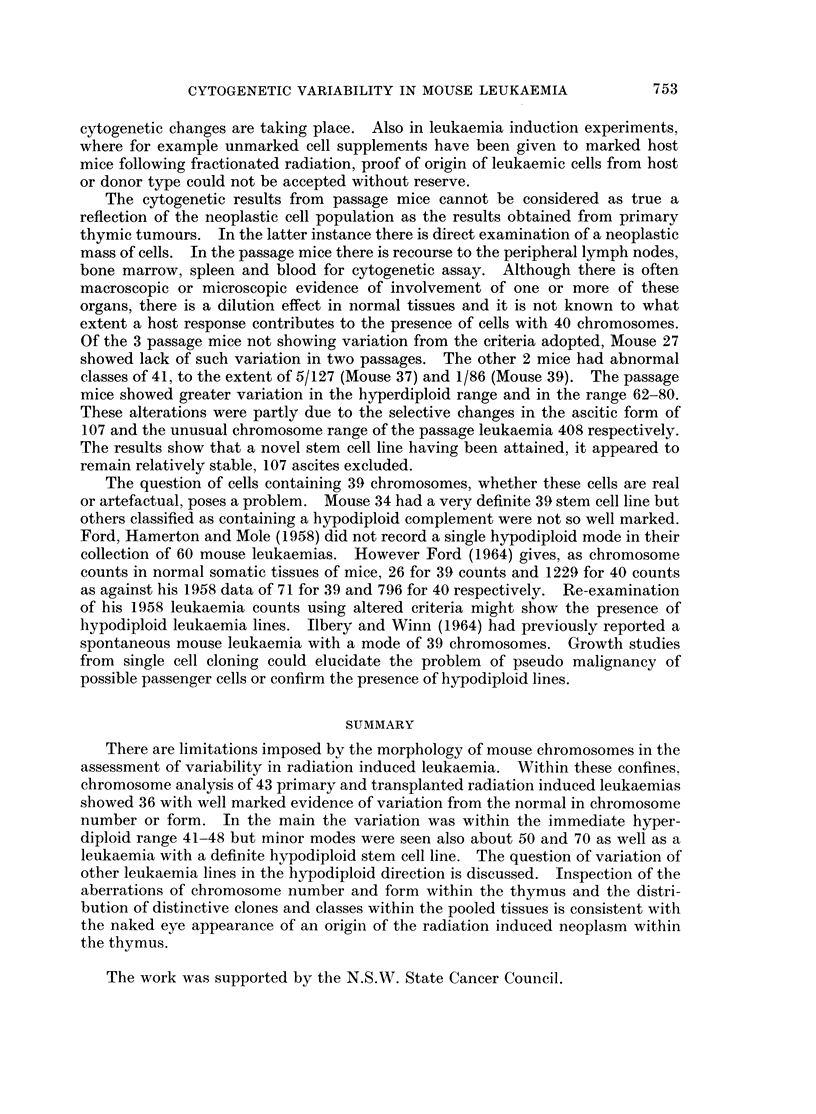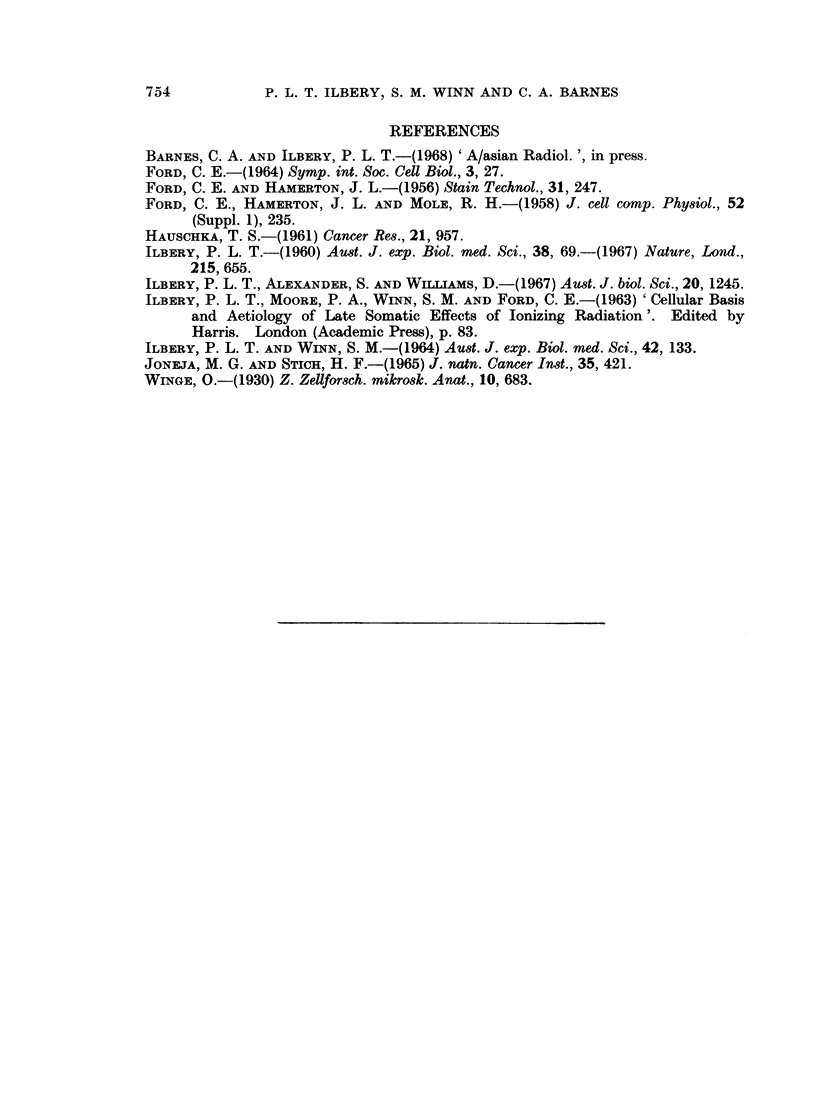# Cytogenetic variability in radiation induced mouse leukaemia.

**DOI:** 10.1038/bjc.1968.88

**Published:** 1968-12

**Authors:** P. L. Ilbery, S. M. Winn, C. A. Barnes

## Abstract

**Images:**


### CYTOGENETIC VARIABILITY IN RADIATION INDUCED MOUSE LEUKAEMIA

P. L. T. ILBERY, S. M. WINN AND C. A. BARNES

*From the School of Public Health and Tropical Medicine, University of Sydney, N.S.W., Australia*

Received for publication June 4, 1968

C57BL mice and certain of their hybrids have a high incidence of radiation induced leukaemia. The incidence is greater than 70% in (C57BL × CBA.T6T6)$F_1$ hybrids aged between 30 and 40 days receiving 4 fractions of 200 rads gamma radiation at 4-day intervals (Ilbery, 1967). The great majority of primary reticular neoplasms arising in irradiated mice show a variation in chromosome number in the range 41 to 45 and distinctive new marker chromosomes are often present (Ford, Hamerton and Mole, 1958). It has been suggested that karyotypic alterations are of primary significance in the onset of neoplasia (Winge, 1930) but more probably there is an association for such changes with tumour progression (Hauschka, 1961). Nevertheless from cytogenetic studies of the thymus in the preleukaemic phase it seems that observable variations in chromosome number and form accompany an early stage of leukaemia induction (Ilbery *et al.*, 1963; Joneja and Stich, 1965).

This report is concerned with the cytogenetic results of mice involved in radiation experiments during the last 5 years and who subsequently exhibited macroscopically leukaemia of the thymic type. Thymomas were passaged so that, where for technical reasons examination of the primary neoplasm failed, subsequent sampling of the malignant cells could be made in the passage mice. Cytogenetic results of a total of 43 radiation induced leukaemias are recorded of which 26 were sampled from the propositi.

A related paper will give the cytogenetic results of mice exposed to the leukaemogenic effects of radiation and in which attempts were made, by the administration of cell supplements, to modify or prevent the onset of leukaemia.

### MATERIALS AND METHODS

Mice of both sexes between 30 and 40 days of age at the time of irradiation were used in the leukaemia induction experiments. The inbred strains employed were C57BL, CBA, DBA and T6T6 all maintained in this laboratory by selective inbreeding during the last 10 years. CBA/H and CBA.T6T6 have been inbred a further 12 and 9 generations respectively in this laboratory since the importation in 1963 of these syngeneic mice from Dr. Mary Lyon of the M.R.C. Radiobiological Research Unit, Harwell.

The source of radiation was radiocobalt. The dose rate varied from 48 to 83 rads/min. at 75 cm. source/target distance. The containers and dosimetry have been previously described (Ilbery, 1960). In 1964 the dose was increased from 4 fractions of 180 rads at 4-day intervals to 200 rads with the same fractionation.

Cytogenetic techniques have evolved during the progress of the experiment from the original Feulgen squash method of Ford and Hamerton (1956) through modifications of the hypotonic, fixative, air drying sequence making use of different stains but latterly using lacto-acetic-orcein (Barnes and Ilbery, 1968). The quality of the preparations has not deteriorated with the advantages accruing from acceleration of the technical processing. The direct method of chromosome preparation entailing the intra-peritoneal injection of Colcemid (7·5 $\mu$g./kg.) and sampling of tissues $1\frac{1}{2}$ hours later has been used throughout the experiments. In scoring aberrations of chromosome number or form in mouse leukaemia it has been our practice (Ilbery *et al.*, 1963) to recognise a clone when more than 5% of the cells examined carried a novel chromosome complement. Also arbitrarily it is considered that a class with an altered chromosome number is present if it is hyperdiploid and constitutes more than 5% of cells. If the class has less than 40 chromosomes then 10% is our requirement for its establishment since spurious counts of less than 40 may arise by artefact in processing and subsequent spreading. It is realised that changes in form are likely to have been underestimated since the uniformly acrocentric nature of mouse chromosomes makes distinction of small changes quite impossible. Metacentric chromosomes and chromosome/chromatid gaps however are very easily distinguishable but unfortunately uncommon in the laboratory's experience in the material to be described. The majority of marker chromosomes enabling the recognition of clones are those translocations and deletions yielding chromosomes larger or smaller than the largest and smallest normal acrocentric mouse chromosomes by a clearly observable amount. In relating changes in length the following symbols from the classification of Ford, Hamerton and Mole (1958) have been used:

| | | | |
|---|---|---|---|
| l | = longest " normal " | s | = shortest " normal " |
| li | ~ 1·20 longest | si | ~ 0·67 shortest |
| lii | ~ 1·50 longest | sii | ~ 0·50 shortest |
| liii | ~ 1·90 longest | siii | ~ 0·25 shortest |

Table I.—*Cytogenetic Details of Pooled Tissues from the Propositi of Radiation of*

| | | Chromosome | | | | | | | | | | | | | | | | | | |
|---|---|---|---|---|---|---|---|---|---|---|---|---|---|---|---|---|---|---|---|---|
| Identification | Strain | <38 | 38 | 39 | 40 | 41 | 42 | 43 | 44 | 45 | 46 | 47 | 48 | 49 | 50 | 51 | 52 | 53 | 54 | 55 |
| 1/15 | C57BL♀ | 1 | 4 | 24 | 14 | 1 | — | — | — | — | — | — | — | — | — | — | — | — | — | — |
| 2/30 | C57BL♀ | 3 | 4 | 8 | 62 | 34 | 281 | 2 | 1 | — | — | — | 1 | — | — | — | — | — | — | — |
| 3/34 | C57BL♂ | — | — | 1 | 25 | 1 | 1 | 1 | — | — | — | — | — | — | — | — | — | — | — | — |
| 4/57 | DBA♂ | 1 | — | 4 | 65 | 1 | 14 | 3 | 1 | — | — | — | — | — | — | — | — | — | — | — |
| 5/112 | (C57BL × T6T6)♂ | — | — | 5 | 87 | 5 | — | — | — | — | — | — | — | — | — | — | — | 1 | 1 | — |
| 6/113 | (C57BL × T6T6)♀ | 3 | — | 5 | 185 | 81 | 2 | 5 | — | — | — | — | — | — | — | — | — | — | — | — |
| 7/116 | (DBA × C57BL)♂ | — | 2 | 2 | 27 | 32 | 114 | 6 | — | 2 | 2 | 1 | 1 | — | — | — | — | — | — | — |
| 8/118 | (T6T6 × C57BL)♀ | 2 | 7 | 42 | 74 | 2 | — | — | — | — | — | — | — | — | — | — | — | — | — | — |
| 9/126 | (T6T6 × C57BL)♂ | 1 | — | 5 | 12 | 21 | 29 | 3 | — | — | — | — | — | — | — | — | — | — | — | — |
| 10/130 | C57BL♀ | — | 13 | 7 | 48 | 2 | 6 | — | — | — | — | — | — | — | — | — | — | — | — | — |
| 11/134 | (C57BL × T6T6)♀ | — | 1 | 1 | 3 | 2 | 4 | 63 | 1 | — | — | — | — | — | — | — | — | — | — | — |
| 12/135 | (T6T6 × C57BL)♀ | 1 | 1 | 1 | 15 | 9 | 51 | 13 | — | — | — | — | — | — | — | — | — | — | — | — |
| 13/140 | (C57BL × T6T6)♂ | — | 1 | 2 | 28 | — | 1 | — | — | — | — | — | — | — | 9 | 10 | 4 | 1 | — | — |
| 14/204 | (C57BL × T6T6)♀ | — | — | 5 | 107 | 21 | 20 | 15 | 8 | — | 1 | — | — | — | — | — | — | — | — | — |
| 15/208 | (57BL/CBA. × T6T6)♀ | — | — | 5 | 172 | 5 | 3 | — | — | — | — | — | — | — | — | — | — | — | — | — |
| 16/230 | C57BL♀ | 2 | 7 | 6 | 56 | 3 | 14 | 9 | 2 | 3 | 6 | — | — | — | — | — | — | — | — | — |
| 17/442 | (C57BL × CBA.T6T6)♀ | — | — | — | 114 | 4 | 3 | — | — | — | — | — | — | — | — | — | — | — | — | — |
| 18/455 | (C57BL × CBA/H)♂ | — | — | — | 5 | 11 | — | — | — | — | — | — | — | — | — | — | — | — | — | — |
| 19/473 | (C57BL × CBA.T6T6)♀ | — | — | — | 44 | 14 | 26 | — | — | — | — | — | — | — | — | — | — | — | — | — |
| 20/493 | (C57BL × CBA/H)♂ | — | — | — | 38 | 2 | — | — | — | — | — | — | — | — | — | — | — | — | — | — |
| 21/514 | (C57BL × CBA.T6T6)♂ | — | — | — | 8 | 19 | 2 | 1 | — | — | — | — | — | — | — | — | — | — | — | — |
| 22/647 | (C57BL × CBA.T6T6)♀ | — | — | — | 15 | — | 5 | 2 | — | — | — | — | — | — | — | — | — | — | — | — |
| 23/650 | (C57BL × CBA.T6T6)♂ | — | 2 | 1 | 22 | 4 | — | — | — | — | — | — | — | — | — | — | — | — | — | — |
| 24/692 | C57BL♀ | — | — | — | 70 | 25 | 9 | 6 | — | — | — | — | — | — | — | — | — | — | — | — |
| 25/710 | (C57BL × CBA/H)♀ | 2 | 3 | 10 | 56 | 22 | 9 | 3 | 2 | — | — | — | — | — | — | — | — | — | — | — |
| 26/714 | (C57BL × CBA.T6T6)♀ | 2 | 1 | 7 | 27 | 2 | 9 | — | — | — | — | — | — | — | — | — | — | — | — | — |

The criterion for accepting mice into the series was that they should have thymomas of probable radiation aetiology. Usually at postmortem involvement in the leukaemic process was also to be seen of lymph nodes, liver and spleen as well as infiltrations in other organs but occasionally a thymoma was the only macroscopic evidence of neoplasia. Thymomas were passaged by homogenisation and intraperitoneal injection of $10^7$ cells. In this way information was available when for one reason or another insufficient or poor-quality metaphases were obtained from the primary tumours.

### RESULTS

The cytogenetic details of 26 primary murine leukaemias of radiation aetiology are given in Table I. The tissues from which the results were aggregated were pooled from two or more of the following tissues: thymus, lymph node, bone marrow, spleen or blood.

Mice 1 to 14 in this table developed leukaemia after a radiation schedule of 4 fractions of 180 rads whole body gamma radiation while the remainder, with the exception of mice 15, 16 and 23 were subjected to 4 fractions of 200 rads whole body gamma radiation. In addition to the radiation schedule Mouse 22 had been given a sleeping dose of amytal prior to each irradiation. Mouse 23 received 3 fractions of 200 rads and a final fraction of 600 rads. Excepting Mouse 16, only mice developing leukaemia within 1 year of irradiation have been included, since the greatest incidence of thymoma is within this time bracket and further, leukaemias appearing after this time are more often of peripheral lymphoid type or reticulum cell sarcoma, rather than the central thymic type. Mouse 16 manifested leukaemia 599 days following neonatal inoculation with cell free extract of radiation induced leukaemia. Thymoma also occurred in a litter mate given the same treatment, its cytogenetic details already having been recorded (Ilbery and Winn, 1964). Mouse 15 was a C57BL radiation chimaera dying 330 days following 925 rads whole body radiation and resuscitation with 5 $\times$ $10^6$

*Induced Leukaemias (Thymic Type)*

Number

| 56 | 57 | 58 | 59 | 60 | 61 | 62 | 63 | 64 | 65 | 66 | 67 | 68 | 69 | 70 | 71 | 72 | 73 | 74 | 75 | 76 | 77 | 78 | 79 | 80 | 81 | 82 | 83 | 84 | Poly |
|---|---|---|---|---|---|---|---|---|---|---|---|---|---|---|---|---|---|---|---|---|---|---|---|---|---|---|---|---|---|
| — | — | — | — | — | — | — | — | — | — | — | — | — | — | — | — | — | — | — | — | — | — | — | — | — | — | — | — | — | — |
| (clone of liii) | | | — | — | — | — | — | — | — | — | — | — | — | — | — | — | — | — | — | — | — | — | 1 | — | — | 3 | — | 1 | 3 |
| — | — | — | — | — | — | — | — | — | — | — | — | — | — | — | — | — | — | — | — | — | — | — | — | 4 | — | — | — | — | — |
| — | — | — | — | — | — | — | — | — | — | — | — | — | — | — | — | — | — | — | — | — | — | — | — | — | — | — | — | — | — |
| (clones of x; ∩; li, lii; sii, li) | | | | | | | — | — | — | — | — | — | — | 3 | 7 | 7 | 41 | 19 | 13 | 5 | 3 | — | — | — | — | — | — | — | 7 |
| — | — | — | — | — | — | — | — | — | — | — | — | — | — | — | — | — | — | — | — | — | — | — | — | — | — | — | — | — | 6 |
| — | — | — | — | — | — | — | — | — | — | — | — | — | — | — | — | — | — | — | — | 3 | — | — | — | 17 | — | 1 | 1 | — | — |
| — | — | — | — | — | — | — | — | — | — | — | — | — | — | — | — | — | — | — | — | — | — | — | — | — | — | — | — | — | 13 |
| — | — | — | — | — | — | — | — | — | — | — | — | — | — | — | — | — | — | — | — | — | — | — | — | — | — | — | — | — | — |
| (clones of ∩; li; si, sii) | | | | | — | — | — | — | — | — | — | — | — | — | — | — | — | — | — | — | — | — | — | — | — | — | — | — | — |
| — | — | — | — | — | — | — | — | — | — | — | — | — | — | — | — | — | — | — | — | — | — | — | — | — | — | — | — | — | — |
| (clone of si) | | | — | — | — | — | — | — | — | — | — | — | — | — | — | — | — | — | — | — | — | — | — | 1 | — | 7 | — | 15 | — |
| — | — | — | — | — | — | — | — | — | — | — | — | — | — | — | — | — | — | — | — | — | — | — | — | — | — | — | — | — | 1 |
| (lii clones—see Table III) | | | | | | — | — | — | — | — | — | — | — | — | — | — | — | — | — | — | — | 4 | — | — | — | — | — | — | 5 |
| (siii; lii; siii, lii clones—see Table II) | | | | | | | | | — | — | — | — | — | — | — | — | — | — | — | — | — | — | — | — | — | — | — | — | — |
| (clone of siii) | | | — | — | 1 | — | — | — | — | 2 | — | — | — | — | — | 1 | — | — | — | — | — | — | — | 1 | — | — | — | — | — |
| (clone of lii) | | | — | — | — | — | — | — | — | — | — | — | — | — | — | — | — | — | — | — | — | — | — | — | — | — | — | — | — |
| — | — | — | — | — | — | — | — | — | — | — | — | — | — | — | — | — | — | — | — | — | — | — | — | — | — | — | — | — | 1 |
| (si in all classes) | | | | — | — | — | — | — | — | — | — | — | — | — | — | — | — | — | — | — | — | — | — | — | — | — | — | — | — |
| — | — | — | — | — | — | — | — | — | — | — | — | — | — | — | — | — | — | — | — | — | — | — | — | — | — | — | — | — | — |
| — | — | — | — | — | — | — | — | — | — | — | — | — | — | — | — | — | — | — | — | — | — | — | — | — | — | — | — | — | — |
| (siii in 43 class) | | | | — | — | — | — | — | — | — | — | — | — | — | — | — | — | — | — | — | — | — | — | — | — | — | — | — | — |
| (x in all thymus cells and clone seen in spleen) | | | | | | | | | | | — | — | — | — | — | — | — | — | — | — | — | — | — | — | — | — | — | — | — |
| — | — | — | — | — | — | — | — | — | — | — | — | — | — | — | — | — | — | — | — | — | — | — | — | — | — | — | — | — | — |
| — | — | — | — | — | — | — | — | — | — | — | — | — | — | — | — | — | — | — | — | — | — | — | — | — | — | — | — | — | — |
| — | — | — | — | — | — | — | — | — | — | — | — | — | — | — | — | — | — | — | — | — | — | — | — | — | — | — | — | — | — |

cells of (CBA $\times$ T6T6)$F_1$ bone marrow. There appeared to have been complete reversion to host type cells as the T6 marker chromosome was not identified. Siii; lii; and siii, lii clones (Fig. 1) were seen in the thymus and the siii, lii clone was seeded in the neighbouring mediastinal and peripheral lymph nodes macroscopically involved, where the clone was seen to be predominant (Table II).

TABLE II.—*Mouse* 15/208. *Cytogenetic Data from a C57BL/(CBA* $\times$ *T6T6) Radiation Chimaera Dying from Leukaemia* 330 *Days Following* 925 *rads* $\gamma$ *Radiation*

| Marker chromosome | 39 | | 40 | | 41 | | 42 | |
|---|---|---|---|---|---|---|---|---|
| | B.M. Th. | Sp. L.N. | B.M. Th. | Sp. L.N. | B.M. Th. | Sp. L.N. | B.M. Th. | Sp. L.N. |
| — | — | — | 43 | 37 | 1 | — | — | — |
| | — | — | 8 | — | 1 | — | 1 | — |
| siii | — | — | — | — | — | — | — | — |
| | — | — | 9 | — | — | — | 1 | — |
| lii | — | — | 2 | 5 | — | — | — | — |
| | — | — | 12 | 3 | 2 | — | — | — |
| siii, lii | — | — | — | — | — | — | — | — |
| | — | 4 | 8 | 45 | — | 1 | 1 | — |

No T6 cells were seen.
(B.M., bone marrow; Sp., spleen; Th., thymus; L.N., lymph node.)

The lii clone was seen to a small extent in lymph node, bone marrow and spleen, while the siii clone was only present in the thymus. Of 43 cells counted from thymus 6 had a chromosome complement greater than 40, these including hyperdiploid classes of the siii; lii; and siii, lii clones.

A (C57BL $\times$ T6T6)$F_1$ hybrid (Mouse 14, Table III) was sacrificed 189 post radiation, the greatly enlarged thymus containing a clone of lii marked cells. Hyperdiploid classes of this clone were present in lymph node, bone marrow and spleen as well as in thymus. Lii clones from which the T6 marker was absent were seen in lymph node, bone marrow and spleen.

A metacentric chromosome was seen in all cells examined from the thymus of Mouse 23 (Fig. 2). A clone containing the metacentric was present in the spleen.

Of the 26 propositi of radiation induced leukaemia, cytogenetic examination of pooled tissues revealed 23 with abnormal chromosomal variation distinguished by the presence of novel clones as defined. Fig. 4 reveals this variation outside the " normal " distribution of counts as represented by the variation observed in the chromosome number of aggregated tissues of thymus, lymph node, bone marrow and spleen in a radiation chimaera experiment (Table VI). In this experiment

EXPLANATION OF PLATE.

FIG. 1.—Chromosome complement of 40 from a thymus cell of Mouse 15/208 showing the presence of marker chromosomes (lii at the periphery about 7 o'clock and siii at the periphery at 12 o'clock).

FIG. 2.—Chromosome set of 39 from a thymus cell of Mouse 23/650, the metacentric marker chromosome being seen to the centre right.

FIG. 3.—The ascitic form of 35/107 showing the distinctive chromosome with an isochromatid gap towards the right at 3 o'clock.

1

2

3

TABLE III.—*Mouse* 14/204. *Cytogenetic Details of a* (*C*57*BL* × *T*6*T*6)$F_1$ *Hybrid Dying from Leukaemia* 189 *Days Following Four Fractions of* 180 *rads* $\gamma$ *Radiation*

| | | <40 | 40 | 41 | 42 | 43 | 44 | 46 | 78 |
|---|---|---|---|---|---|---|---|---|---|
| Thymus | T6 | — | 2 | 2 | 1 | 1 | — | — | — |
| | T6− | — | — | — | — | — | — | — | — |
| | T6, lii | — | 14 | 3 | 2 | 3 | — | — | — |
| | T6−, lii | — | — | — | — | — | — | — | — |
| Lymph node | T6 | 1 | 2 | 2 | 1 | 2 | — | — | 2 |
| | T6− | — | 1 | 1 | 1 | 1 | — | — | — |
| | T6, lii | 1 | 11 | 8 | 6 | 5 | — | — | 2 |
| | T6−, lii | 1 | — | — | 1 | — | — | — | — |
| Bone marrow | T6 | — | 1 | — | — | — | — | — | — |
| | T6− | — | — | — | — | — | — | — | — |
| | T6, lii | — | 36 | — | 5 | 1 | 3 | — | — |
| | T6−, lii | — | 7 | — | — | — | 1 | — | — |
| Spleen | T6 | 2 | 9 | 1 | — | — | 1 | — | — |
| | T6− | — | 7 | — | — | 1 | 1 | — | — |
| | T6, lii | — | 14 | 3 | 2 | 1 | 1 | 1 | — |
| | T6 , lii | — | 3 | 1 | 1 | — | 1 | — | — |

(C57BL × CBA.T6T6)$F_1$ mice had been exposed to a lethal dose of 1050 rads gamma whole body radiation but with a single lymph node excluded and then given 6 × $10^6$ cells of (C57BL × CBA/H)$F_1$ bone marrow by the intravenous route. The majority of the cells counted therefore represent an actively dividing colonising population of normal cells derived from bone marrow (T6−) and a lesser number presumably derived from lymph node (T6+).

Sixteen thymomas were observed cytogenetically. Only 1 (6, Table IV) did not show the presence of a novel clone as defined but a single hyperdiploid cell was seen in 34 thymus cells counted. An hyperdiploid 41 class was present in other tissues. The variation of the thymoma material about the " normal " distribution is shown in Fig. 4. The distribution of chromosome counts in the aggregated tissues of the propositi (Fig. 5) closely mirrors the thymoma distribution. However, the variation in the chromosome count range of 70–84 was not seen in this thymoma sample. Fig. 5 illustrates the marked increase in cytogenetic variation in aggregated tissues of passage mice both in the hyperdiploid direction and in the extent of the near tetraploid variation.

The cytogenetic details of the 17 passage mouse leukaemias of radiation aetiology are given in Table V. The primary thymic tumours had been passaged intraperitoneally. Usually evidence of successful neoplastic passage in the injected mice was to be found in enlargement of the liver, spleen and lymph nodes. Subsequent passages were made by homogenisation of spleen and further intraperitoneal transfer, except for the ascites and solid form of 107. These were passaged as ascites intraperitoneally and by homogenisation of the solid tumour and subcutaneous injection respectively. Examination of pooled tissues showed that 5 passage leukaemias appeared to be hypodiploid, while 3 were diploid and 9 hyperdiploid. Mouse 81 had a very definite 39 stem cell line in passage 4 but either a dilution effect or selection changed the stem cell line in later passages. Selection is well seen in 107. At the second intraperitoneal spleen passage an ascitic form appeared with a modal number of 42. A distinctive chromosome with an isochromatid gap was present (Fig. 3). This marker chromosome was observed in many cells up to passage 8 and in an occasional cell up to passage 16. The stem cell line became more hyperdiploid up to passage 16 and remained about a mode of 45 in the two later passages. In contrast the solid form of the tumour, arising from subcutaneous passage of the first ascitic form and running in parallel passage, retained the distinctive marker into the 47th passage and did not show as much class variation as the ascitic form.

FIG. 4.—Log. frequency per thousand cells of chromosome counts of various classes. Distribution of chromosome counts:

– – – – – – – – ; normal tissues of mice in a radiation transplantation experiment (Table VI).
× ———— × ; thymomas of the propositi from the data given in Table IV.

### DISCUSSION

Assessment of variation in the mouse sets of chromosomes is limited to alterations in number and to those changes in form which can be distinguished in a karyotype consisting of acrocentric chromosomes differing in size over a spectral range of about 3. Metacentrics and chromatid/chromosome gaps are uncommon so that the examination is limited mostly to those aberrations in form having major departures from the shortest or longest chromosome length. Thus a chromosome count of 40 in the mouse cannot imply indubitably that a cell has a

TABLE IV.—*Cytogenetic Details of Thymus from the Propositi of Radiation Induced Leukaemia (Thymic Type)*

| Identification | Strain | Chromosome Number | | | | | | | | | | | | | | |
|---|---|---|---|---|---|---|---|---|---|---|---|---|---|---|---|---|
| | | 39 | 40 | 41 | 42 | 43 | 44 | 45 | 46 | 47 | 48 | 49 | 50 | 51 | 52 | 53 |
| 2/30 | C57BL♀ | 2 | 1 | — | 45 | — | — | — | — | — | — | — | — | — | — | — |
| 4/57 | DBA♂ | — | — | — | 13 | — | — | — | — | — | — | — | — | — | — | — |
| 6/113 | (C57BL × T6T6)♀ | — | 34 | 1 | — | — | — | — | — | — | — | — | — | — | — | — |
| 7/116 | (DBA × C57BL)♂ | — | — | — | 7 | — | — | — | — | — | — | — | — | — | — | — |
| 8/118 | (T6T6 × C57BL)♀ | 19 | 21 | — | — | — | — | — | — | — | — | — | — | — | — | — |
| 9/126 | (T6T6 × C57BL)♂ | — | 4 | 2 | 13 | 4 | — | — | — | — | — | — | — | — | — | — |
| 10/130 | C57BL♀ | 2 | 27 | 1 | 6 | — | — | — | (si; 2∩) | | — | — | — | — | — | — |
| 12/135 | (T6T6 × C57BL)♀ | 1 | 1 | 3 | 16 | 13 | — | — | (si) | — | — | — | — | — | — | — |
| 13/140 | (C57BL × T6T6)♂ | — | — | — | — | — | — | — | — | — | — | — | 9 | 10 | 4 | 1 (sii; 2sii) |
| 14/204 | (C57BL × T6T6)♀ | — | 16 | 5 | 3 | 4 | — | — | — | (lii)—see Table III | | | | | — | — |
| 15/208 | C57/(CBA × T6T6)♀ | — | 35 | 3 | 3 | — | — | — | (siii; lii; siii, lii)—see Table II | | | | | | | — |
| 16/230 | C57BL♀ | 1 | 10 | 1 | 3 | 1 | 1 | — | — | — | — | — | — | — | — | — |
| 17/442 | (C57BL × CBA.T6T6)♀ | — | 28 | 4 | 3 | — | — | — | (lii) | — | — | — | — | — | — | — |
| 23/650 | (C57BL × CBA.T6T6)♂ | 1 | 5 | 4 | — | — | — | — | (× in all)— | | | — | — | — | — | — |
| 24/692 | C57BL♀ | — | 12 | 4 | 4 | 5 | — | — | — | — | — | — | — | — | — | — |
| 26/714 | (C57BL × CBA.T6T6)♀ | 2 | 16 | — | 6 | — | — | — | — | — | — | — | — | — | — | — |

(The presence of clones is indicated within brackets.)

FIG. 5.—Log. frequency per thousand cells of chromosome counts of various classes. Distribution of Chromosome counts:

+———+ ; aggregated tissues of the propositi from the data given in Table I.
○———○ ; aggregated tissues of passage leukaemic mice (Table V).

TABLE V.—*Cytogenetic Details of Radiation Induced Leukaemias Maintained for*

| Identification | Strain | Passage | Chromosome <38 | 38 | 39 | 40 | 41 | 42 | 43 | 44 | 45 | 46 | 47 | 48 | 49 |
|---|---|---|---|---|---|---|---|---|---|---|---|---|---|---|---|
| 27/6 | C57BL♀ | 1 | — | — | — | 27 | — | — | — | — | — | — | — | — | — |
| ,, | ,, | 2 | — | 2 | 3 | 66 | — | — | — | — | — | — | — | — | — |
| 28/9 | C57BL♀ | 1 | 1 | — | — | 29 | — | — | — | — | — | — | — | — | — |
| ,, | ,, | 5 | — | 7 | 8 | 48 | 4 | — | — | — | — | — | — | — | — |
| 29/19 | C57BL♀ | 1 | 1 | 8 | 3 | 60 | 2 | — | — | — | — | — | — | — | — |
| 30/20 | C57BL♀ | 1 | — | — | 2 | 19 | — | — | — | — | — | — | — | — | — |
| ,, | ,, | 3 | — | 1 | 17 | 55 | — | — | — | — | — | — | — | — | — |
| 31/41 | (CBA × C57BL)♀ | 3 | — | — | 1 | 19 | 3 | 19 | 10 | 1 | 1 | — | — | — | — |
| ,, | ,, | 20 | — | — | — | 39 | 4 | 31 | 12 | — | — | — | — | — | — |
| 32/53 | C57BL♀ | 3 | — | 2 | 12 | 47 | — | — | — | — | — | — | — | — | — |
| 33/64 | C57BL♀ | 1 | — | — | — | 2 | 8 | 3 | — | — | — | — | — | — | — |
| 34/81 | C57BL♂ | 4 | — | 2 | 44 | 13 | — | — | — | — | — | — | — | — | — |
| ,, | ,, | 10 | — | 1 | 16 | 20 | — | — | — | — | — | — | — | — | — |
| ,, | ,, | 13 | — | 2 | 21 | 21 | 2 | — | — | — | — | — | — | — | — |
| 35/107 | CBA♂ | Ascites 2 | — | — | — | 1 | 3 | 94 | 19 | — | — | — | — | — | — |
| ,, | ,, | ,, 3 | — | — | — | 2 | 2 | 49 | 28 | 5 | — | — | — | — | — |
| ,, | ,, | ,, 8 | — | 1 | 4 | 3 | 16 | 22 | 15 | 1 | 1 | — | — | — | — |
| ,, | ,, | ,, 10 | — | — | — | — | 1 | 13 | 20 | 16 | 1 | 2 | — | — | — |
| ,, | ,, | ,, 13 | — | — | 3 | 5 | 2 | 1 | 14 | 2 | 12 | 2 | 1 | — | 1 |
| ,, | ,, | ,, 16 | — | — | — | — | — | — | 11 | 23 | 60 | 8 | — | — | — |
| ,, | ,, | ,, 17 | — | — | 1 | 2 | — | 2 | 47 | 22 | 54 | 17 | 8 | — | — |
| ,, | ,, | ,, 18 | — | — | — | 3 | 1 | 2 | 9 | 19 | 31 | 9 | 1 | — | — |
| ,, | ,, | Solid 4 | — | — | — | — | — | 1 | 70 | 1 | 1 | — | — | — | — |
| ,, | ,, | ,, 12 | — | — | — | — | — | — | 30 | — | — | — | — | — | — |
| ,, | ,, | ,, 14 | — | — | — | — | — | — | 3 | — | — | — | — | — | — |
| ,, | ,, | ,, 36 | — | — | — | — | — | 1 | 7 | 2 | — | — | — | — | — |
| ,, | ,, | ,, 47 | — | — | — | 1 | 3 | 6 | 13 | 8 | 4 | 2 | — | — | — |
| 36/143 | C57BL♀ | 3 | — | — | — | — | — | 2 | 1 | 37 | — | — | — | — | — |
| 37/191 | C57BL♀ | 3 | — | 1 | 8 | 113 | 5 | — | — | — | — | — | — | — | — |
| 38/194 | C57BL♂ | 1 | — | — | 3 | 82 | 1 | — | — | — | — | — | — | — | — |
| 39/205 | C57BL♀ | 1 | — | — | 6 | 91 | 27 | — | — | — | — | — | — | — | — |
| 40/290 | (C57BL × T6T6)♀ | 1 | — | — | — | 7 | — | — | — | — | — | — | — | — | — |
| 41/332 | C57BL♀ | 6 | — | 2 | 26 | 74 | — | — | — | — | — | — | — | — | — |
| 42/379 | C57BL♀ | 6 | 1 | 1 | 2 | 51 | 1 | — | — | 1 | 2 | 12 | 7 | 42 | 22 |
| ,, | ,, | 7 | — | — | — | 21 | 1 | — | — | — | — | 3 | — | 33 | — |
| ,, | ,, | 8 | — | — | — | — | — | — | — | — | — | — | 1 | 9 | 4 |
| ,, | ,, | 9 | — | — | — | 1 | — | — | — | — | — | — | 1 | 2 | — |
| 43/408 | (C57BL × T6T6)♀ | 1 | — | 2 | 1 | 10 | 1 | — | — | — | — | — | — | — | — |
| ,, | ,, | 3 | — | — | 1 | — | — | — | — | — | — | — | — | — | — |

normal chromosome set. Further, cryptic changes may include cells with 40 chromosomes apparently normal in form but actually altered in chromosome complement. For example non-disjunction may give a cell with 39 chromosomes; another such event in the same cell may then yield a cell containing 40 chromosomes but with trisomy for one chromosome pair which would pass entirely unnoticed. It would be possible to detect these hidden changes in a marsupial with a dozen distinctive chromosomes or in the hamster. Yet the established yield of leukaemia in the mouse from a given radiation schedule, the mouse's inbred strains and its built-in chromosome marker in the CBA.T6T6 line favour its continued use for the present.

Within these confines the outstanding impression gained from this mouse leukaemia material obtained by direct examination of tissues is the extent of the aberrations of chromosome number and form readily visible. There is a contrasting almost complete lack of variability in the cytogenetic data accumulated in this laboratory from direct examination of normal humans, sheep, goat, oxen, marsupials and rats as well as mice. Table VI scores cells from thymus, lymph node, bone marrow and spleen for the presence of the T6 marker in a recent mouse radiation chimaera experiment in which rapid proliferation of normal tissues occurs. It can be seen that less than 1 % of cells are recorded with either more or less than the standard chromosome set of 40. Chromosome/chromatid breaks are occasionally seen in short-term cultures and a clone was seen to arise in a long-term fibroblast culture (Ilbery, Alexander and Williams, 1967) but aberrations of form are rare in direct examinations of normal tissue.

*One or More Passages*

**Number**

| 50 | ~ | 59 | 60 | 61 | 62 | 63 | 64 | 65 | 66 | 67 | 68 | 69 | 70 | 71 | 72 | 73 | 74 | 75 | 76 | 77 | 78 | 79 | 80 | 81 | 82 | 83 | 84 | 85 | 86 | 87 | 88 | 89 | 90 | Poly |
|---|---|---|---|---|---|---|---|---|---|---|---|---|---|---|---|---|---|---|---|---|---|---|---|---|---|---|---|---|---|---|---|---|---|---|
| — | — | — | — | — | — | — | — | — | — | — | — | — | — | — | — | — | — | — | — | — | — | — | — | — | — | — | — | — | — | — | — | — | — | — |
| — | — | — | — | — | — | — | — | — | — | — | — | — | — | — | — | — | — | — | — | — | — | — | — | — | — | — | — | — | — | — | — | — | — | — |
| — | — | 1 | — | — | — | — | — | — | — | — | — | — | — | — | — | — | — | — | — | — | 1 | 1 | 9 | 2 | 1 | — | — | — | — | — | — | — | — | — |
| — | — | 7 | — | — | — | — | — | — | — | — | — | — | — | — | — | — | — | — | — | — | 2 | 3 | 9 | 3 | 6 | — | — | — | — | — | — | — | — | 2 |
| (∩) | — | — | — | — | — | — | — | — | — | — | — | — | — | — | — | — | — | — | — | — | — | — | — | — | — | — | — | — | — | — | — | — | — | — |
| — | — | — | — | — | — | — | — | — | — | — | — | — | — | — | — | — | — | — | — | — | — | — | — | — | — | — | — | — | — | — | — | — | — | — |
| — | — | — | — | — | — | — | — | — | — | — | — | — | — | — | — | — | — | — | — | — | 1 | — | — | — | — | — | — | — | — | — | — | — | — | — |
| — | — | — | — | — | — | — | — | — | — | — | — | — | — | — | — | — | — | — | — | — | — | — | — | — | — | — | — | — | — | — | — | — | — | — |
| 1 | — | — | — | — | — | — | — | — | — | — | — | — | — | — | — | — | — | — | 1 | — | — | — | 1 | — | 2 | — | 4 | — | 1 | — | — | — | — | — |
| — | — | — | — | — | — | — | — | — | — | — | — | — | — | — | — | — | — | — | — | — | — | — | — | — | — | — | — | — | — | — | — | — | — | — |
| — | — | — | — | — | — | — | — | — | — | — | — | — | — | — | — | — | — | — | — | — | — | — | — | — | — | — | — | — | — | — | — | — | — | — |
| — | — | — | — | — | — | — | — | — | — | — | — | — | — | — | — | — | — | — | — | — | 1 | — | 1 | — | — | — | — | — | — | — | — | — | — | — |
| — | — | — | — | — | — | — | — | — | — | — | — | — | — | — | — | — | — | — | — | 1 | — | — | — | — | — | — | — | — | — | — | — | — | — | 12 |
| — | — | — | — | — | — | — | — | — | — | — | — | — | — | — | — | — | — | — | — | — | 1 | — | — | — | — | — | — | — | — | — | — | — | — | — |
| (∩) | — | — | — | — | — | — | — | — | — | — | — | — | — | — | — | — | — | — | — | — | — | — | — | — | — | — | — | — | — | — | — | — | — | — |
| (all 43 class have si, 67 with ∩) | | | | | | | — | — | — | — | — | — | — | — | — | — | — | — | — | — | — | — | — | — | — | — | — | — | — | — | — | — | — | — |
| (no si, 5x, all ∩) | | | | — | — | — | — | — | — | — | — | — | — | — | — | — | — | — | — | — | — | — | — | — | — | — | — | — | — | — | — | — | — | — |
| (si; x) | | — | — | — | — | — | — | — | — | — | — | — | — | — | — | — | — | — | — | — | — | — | — | — | — | — | — | — | — | — | — | — | — | — |
| — | — | — | — | — | — | — | — | — | — | — | — | — | — | — | — | — | — | — | 1 | — | — | — | — | — | — | — | — | 1 | 2 | — | — | 1 | — | — |
| (x; 2si; siii; and occasional ∩) | | | | | | | — | — | — | — | — | — | — | — | — | — | — | — | — | — | — | — | — | — | — | — | — | — | — | 1 | — | — | 1 | 1 |
| (si; 2si; 3si; 2si. siii; siii) | | | | | | — | — | — | — | — | — | — | — | — | — | — | — | — | — | — | — | — | — | — | — | — | — | — | — | — | — | — | 1 | — |
| (si; 3si; li. si; siii; 2siii) | | | | | | — | — | — | — | — | — | — | — | — | — | — | — | — | — | — | — | — | — | — | — | — | — | — | — | — | — | — | 1 | — |
| (∩) | — | — | — | — | — | — | — | — | — | — | — | — | — | — | — | — | — | — | — | — | — | — | — | — | — | — | — | — | — | — | — | — | — | — |
| (∩) | — | — | — | — | — | — | — | — | — | — | — | — | — | — | — | — | — | — | — | — | — | — | — | — | — | — | — | — | — | — | — | — | — | — |
| (∩) | — | — | — | — | — | — | — | — | — | — | — | — | — | — | — | — | — | — | — | — | — | — | — | — | — | — | — | — | — | — | — | — | — | — |
| (li and ∩) | | | — | — | — | — | — | — | — | — | — | — | — | — | — | — | — | — | — | — | — | — | — | — | — | — | — | — | — | — | — | — | — | — |
| (∩∩; , li∩∩; ) | | | | — | — | — | — | — | — | — | — | — | — | — | — | — | — | — | — | — | — | — | — | — | — | — | — | — | — | — | — | — | — | — |
| (3siii) | | — | — | — | — | — | — | — | — | — | — | — | — | — | — | — | — | — | — | — | — | — | — | — | — | — | — | — | — | — | — | — | — | — |
| — | — | — | — | — | — | — | — | — | — | — | — | — | — | — | — | — | — | — | — | — | — | — | 1 | — | — | — | — | — | — | — | — | — | — | — |
| — | — | — | — | — | — | — | — | — | — | — | — | — | — | — | — | — | — | — | — | — | — | — | — | — | — | — | — | — | — | — | — | — | — | — |
| (si; sii; si, sii; 2sii in most cells) | | | | | | | — | — | — | — | — | — | — | — | — | — | — | — | — | — | — | — | — | — | — | — | — | — | — | — | — | — | — | — |
| — | — | 1 | 2 | — | — | — | 1 | 1 | — | 1 | — | — | — | — | — | — | — | — | — | — | 1 | (si sii; sii) | | | | — | — | — | — | — | — | — | — | — |
| — | — | — | — | — | — | — | — | — | — | — | — | — | — | — | — | — | — | — | — | — | — | — | — | — | — | — | — | — | — | — | — | — | — | — |
| 3 | — | 1 | — | — | — | — | — | — | — | — | — | — | — | — | — | — | — | — | — | — | — | — | 3 | — | — | — | — | — | — | — | — | — | — | 1 |
| — | — | — | — | — | — | — | — | — | — | — | — | — | — | — | — | — | — | — | — | — | — | — | — | — | — | — | — | — | — | — | — | — | — | — |
| 1 | — | — | — | — | — | — | — | — | — | — | — | — | — | — | — | — | — | — | — | — | — | — | — | — | — | — | — | — | — | — | — | — | — | — |
| 1 | — | — | — | — | — | — | — | — | — | — | — | — | — | — | — | — | — | — | — | — | — | — | — | — | — | — | — | — | — | — | — | — | — | — |
| — | — | — | — | — | — | 11 | 2 | 4 | 6 | 4 | 11 | 10 | 16 | 15 | 10 | 7 | 6 | 6 | 4 | 2 | 1 | 3 | 1 | — | — | — | — | — | — | — | — | 1 | — | — |
| — | — | — | — | — | — | — | 1 | 1 | — | — | 2 | 3 | 2 | 4 | 2 | 5 | 4 | 5 | 1 | — | — | — | — | — | — | — | — | — | — | — | — | — | — | — |

(Symbols within brackets refer to clones as defined)

TABLE VI.—*Variation in Chromosome Number of Aggregated Tissues* in a Radiation Chimaerism Experiment*

| | 38 | 39 | 40 | 41 | 42 | 43 | 44 | 45 | 46 | Poly |
|---|---|---|---|---|---|---|---|---|---|---|
| T6+ | 1 | 1 | 234 | 9 | 1 | .. | .. | .. | .. | .. |
| T6− | .. | 2 | 3167 | 6 | 1 | .. | .. | .. | .. | 15 |
| | 1 | 3 | 3401 | 15 | 2 | .. | .. | .. | 1 | 15 |

* Thymus, lymph node, bone marrow and spleen.

Accepting the wide criteria for normality for the purpose of these leukaemia experiments of 5% for cells containing more than 40 chromosomes and 10% for less than 40 chromosomes, then abnormal clones or classes have been seen in 22 out of 26 primary leukaemias and in 14 out of 17 passage leukaemias. Their presence may well be more universal in leukaemic tissue than shown. Although examination of aggregated tissues (Table I) did not demonstrate clearly the presence of clones or classes other than 40 in Mice 3, 15, 17 and 20, Mice 15 and 17 both have hyperdiploid clones or classes when the thymus alone is considered (Table IV). The thymus is the tissue in which novel clones have been found more commonly and thymic results were not obtained for Mice 3 and 20. Thus it is not certain that clones or classes were not present in all thymomas.

Whether these chromosome aberrations are epiphenomena depends *inter alia* on when the cell is judged to be neoplastic. The amount of variation taking place has been estimated in the induction period following killing of cells by radiation. At that time an increased rate of cell replacement might be expected in an altered immunological environment. Between 113 and 183 days following a leukaemogenic schedule of irradiation and before the thymus was more than 70 mg. in weight and clinically enlarged, novel clones were shown to be present in the thymus in 12 out of 19 mice (Ilbery *et al.*, 1963). Certainly the cells present in these thymuses might be considered preleukaemic as they failed to transplant. The variation within the cells of the thymus was not matched by variation in the other tissues examined—lymph gland, bone marrow and spleen.

All mice exposed to a leukaemogenic schedule of radiation had thymomas whether or not peripheral lymphoid tissue, liver or spleen were involved. In all but 1 of the 16 primary thymomas examined cytogenetically (Table IV) there was extensive evidence of variation from the normal mode. In this one instance 1 out of 34 cells in the thymus contained 41 chromosomes, the 41 class being abundant in lymph node, bone marrow and spleen. With this possible exception, inspection of the aberrations of chromosome number and form within the thymus and the distribution of distinctive clones and classes within the pooled tissues are consistent with an origin of the radiation induced neoplasm within the thymus. Such an origin is supported by accumulated macroscopic and histological evidence. Specifically cytogenetic analysis of Mouse 15 (Table III) suggests an origin of an lii clone in the thymus with seeding in lymph node, bone marrow and spleen. Again the metacentric chromosome seen in all thymic cells of Mouse 23 was present as a clone within the spleen. The theme of a thymic origin of the neoplastic cell is expanded in the second part of the paper.

Loss of the T6 marker chromosome (Mouse 14, Table III) has important implications. It means that reliance on the presence of T6 as a marker cannot invariably be made in leukaemia induction experiments because selective serial cytogenetic changes are taking place. Also in leukaemia induction experiments, where for example unmarked cell supplements have been given to marked host mice following fractionated radiation, proof of origin of leukaemic cells from host or donor type could not be accepted without reserve.

The cytogenetic results from passage mice cannot be considered as true a reflection of the neoplastic cell population as the results obtained from primary thymic tumours. In the latter instance there is direct examination of a neoplastic mass of cells. In the passage mice there is recourse to the peripheral lymph nodes, bone marrow, spleen and blood for cytogenetic assay. Although there is often macroscopic or microscopic evidence of involvement of one or more of these organs, there is a dilution effect in normal tissues and it is not known to what extent a host response contributes to the presence of cells with 40 chromosomes. Of the 3 passage mice not showing variation from the criteria adopted, Mouse 27 showed lack of such variation in two passages. The other 2 mice had abnormal classes of 41, to the extent of 5/127 (Mouse 37) and 1/86 (Mouse 39). The passage mice showed greater variation in the hyperdiploid range and in the range 62–80. These alterations were partly due to the selective changes in the ascitic form of 107 and the unusual chromosome range of the passage leukaemia 408 respectively. The results show that a novel stem cell line having been attained, it appeared to remain relatively stable, 107 ascites excluded.

The question of cells containing 39 chromosomes, whether these cells are real or artefactual, poses a problem. Mouse 34 had a very definite 39 stem cell line but others classified as containing a hypodiploid complement were not so well marked. Ford, Hamerton and Mole (1958) did not record a single hypodiploid mode in their collection of 60 mouse leukaemias. However Ford (1964) gives, as chromosome counts in normal somatic tissues of mice, 26 for 39 counts and 1229 for 40 counts as against his 1958 data of 71 for 39 and 796 for 40 respectively. Re-examination of his 1958 leukaemia counts using altered criteria might show the presence of hypodiploid leukaemia lines. Ilbery and Winn (1964) had previously reported a spontaneous mouse leukaemia with a mode of 39 chromosomes. Growth studies from single cell cloning could elucidate the problem of pseudo malignancy of possible passenger cells or confirm the presence of hypodiploid lines.

### SUMMARY

There are limitations imposed by the morphology of mouse chromosomes in the assessment of variability in radiation induced leukaemia. Within these confines, chromosome analysis of 43 primary and transplanted radiation induced leukaemias showed 36 with well marked evidence of variation from the normal in chromosome number or form. In the main the variation was within the immediate hyper-diploid range 41–48 but minor modes were seen also about 50 and 70 as well as a leukaemia with a definite hypodiploid stem cell line. The question of variation of other leukaemia lines in the hypodiploid direction is discussed. Inspection of the aberrations of chromosome number and form within the thymus and the distribution of distinctive clones and classes within the pooled tissues is consistent with the naked eye appearance of an origin of the radiation induced neoplasm within the thymus.

The work was supported by the N.S.W. State Cancer Council.

### REFERENCES

Barnes, C. A. and Ilbery, P. L. T.—(1968) ' A/asian Radiol. ', in press.
Ford, C. E.—(1964) *Symp. int. Soc. Cell Biol.*, **3**, 27.
Ford, C. E. and Hamerton, J. L.—(1956) *Stain Technol.*, **31**, 247.
Ford, C. E., Hamerton, J. L. and Mole, R. H.—(1958) *J. cell comp. Physiol.*, **52** (Suppl. 1), 235.
Hauschka, T. S.—(1961) *Cancer Res.*, **21**, 957.
Ilbery, P. L. T.—(1960) *Aust. J. exp. Biol. med. Sci.*, **38**, 69.—(1967) *Nature, Lond.*, **215**, 655.
Ilbery, P. L. T., Alexander, S. and Williams, D.—(1967) *Aust. J. biol. Sci.*, **20**, 1245.
Ilbery, P. L. T., Moore, P. A., Winn, S. M. and Ford, C. E.—(1963) ' Cellular Basis and Aetiology of Late Somatic Effects of Ionizing Radiation '. Edited by Harris. London (Academic Press), p. 83.
Ilbery, P. L. T. and Winn, S. M.—(1964) *Aust. J. exp. Biol. med. Sci.*, **42**, 133.
Joneja, M. G. and Stich, H. F.—(1965) *J. natn. Cancer Inst.*, **35**, 421.
Winge, O.—(1930) *Z. Zellforsch. mikrosk. Anat.*, **10**, 683.